# Editorial

**Published:** 1903-01-15

**Authors:** 


### EDITORIAL.

WITH this issue the DENTAL REGISTER begins the fifty-seventh year of its existence. This is a long life for a professional periodical of any kind. That the existence of this journal has been prolonged to this extent is due in part to the fact that it has always had a mission and has labored to fulfill that mission in as perfect a manner as circumstances allowed. Very much credit is due to the persistent determination of its former editors, that it should be maintained whatever the sacrifice of time and money, in spite of every adverse circumstance. While it was organized as the official organ of Western Dental Societies, it has always given a portion of its space to national affairs. It has been the chief desire of the present management to place on permanent record the transactions of the several dental societies which are located in the territory which it serves, and a consultation of its pages for the past twelve months will, we believe, disclose the fact that it has made a creditable showing, as compared with other journals, in the publication of original matter of practical interest and value to the profession. It is our purpose to continue this policy for the ensuing year; and we are arranging for and expect to add the proceedings of other societies to those which we now regularly report. We shall also publish a comment at least on the transactions of all the national meetings. In addition to this we shall reprint such articles of value as may appear in other periodicals to which our constituents are not likely to have access; and from four to six pages in each issue, of short excerpts of practical importance. We can only promise to carry out our program, providing we have the cordial sympathy and co-operation of our readers, which we confidently expect.

---

The thirty-seventh annual meeting of the Ohio State Dental Society, was held at Columbus, December 2d to 4th, and was one of the best meetings of this society. The literary program was of a high order of merit, and received careful consideration. The attendance was not so large as it should have been, considering the number of dentists practicing in the state. It would seem that there ought to be at least five hundred dentist in the state of Ohio who should be sufficiently interested to attend this meeting and make such contributions as they might be able to do to its deliberations. There were dentists present from considerable distances, as far as Chicago and Philadelphia. If such men can spare of their valuable time to attend these meetings and take part in the proceedings, it would seem that practitioners within the state ought to have sufficient interest to at least come out and listen to the proceedings, and see the exhibits of clinics and supplies which at this meeting were of such interest that they alone would pay for the expenses involved in the trip. It is always a matter of regret to those who habitually attend society meetings that more of the members do not take part in the proceedings of our societies. There is no one who can not out of his experience add something to the general good of his calling if he will only put his pride in his pocket, and take his part in the clinics or discussions.

The Odontographic Society of Chicago, is making great plans to celebrate its fifteenth anniversary, by holding one of the largest dental meetings ever convened on this continent. The meetings are called for February 16th and 17th. Papers will be read by four distinguished men, and nearly two hundred clinics will be given. The clinicians will be from nearly every state in the union, and it is expected that two thousand dentists will be in attendance. We recently spent a week in Chicago and scarcely any other topic was so frequently mentioned as the great meeting. Every one who cares to come is invited, and preparations are being made to interest every one who comes. The meetings and clinics will be held in the commodious rooms of the Northwestern Dental School, which are admirably adapted to the purpose.

The tenth annual meeting of the Institute of Dental Pedagogics, was held at the Palmer House in Chicago, December 29th to 31st. More than thirty colleges were represented by from one to five or six delegates. Three sessions were held each day, except the last one, on which but two sessions were held. This is one of the dental societies which does more conscientious and systematic work than any other organization with which we are acquainted. Its proceedings are full of interest especially to teachers, and no time is wasted in irrelevant discussions of mediocre papers or subjects. Its papers are planned for a year in advance, thus giving their authors ample time to mature papers of sufficient interest and value to claim the most serious consideration. In view of the fact that the college course has now been extended to four years, the trend of papers at this meeting was along the line of the new curriculum. Almost every subject discussed was considered in its relations to this advance. By some members of the profession it is thought that there is not enough in dentistry to require four years of college preparation; but it was very evident to every one present at this meeting that real advances in instruction are being planned for, and the principle solicitude seem to be to make a selection of things most needed in order that the students might not be overworked. The general practitioner does not realize so clearly as the teachers in our colleges that the requirements of the dentist of to-day are so expanded that if graduates of our schools are to take the highest places they must be better educated than ever before. To accomplish this more time and better qualifications for admission are absolutely necessary. If we could require an academic training to start with it would be possible perhaps in three years to accomplish the technical training necessary. Since this seems to be impracticable, nothing remains but for the professional schools to do the work which by right ought to be done in preparatory schools. The advances which are likely to be made in instruction will probably be in the direction physics, orthodontia, porcelain work, oral surgery and therapeutics. As the deliberations of this organization are only advisory no definite action could be taken on the subject of a curriculum for the colleges to adopt, but the committee on curriculum submitted with its report a curriculum which is to be printed and sent to each school, for publication in the announcements, which must be printed before the next meeting of the National Association of Dental Faculties. It is scarcely expected that this curriculum will meet the approval of all schools, but it is hoped that it may at least furnish a bases upon which each school can form its own curriculum.

